# Dose-dependent effects of pomegranate peel extract on modulating ruminal fermentation, methane emission, nutrient digestibility and productive values in camels: an *in vitro* and *in silico* integrations

**DOI:** 10.3389/fvets.2026.1769637

**Published:** 2026-05-04

**Authors:** Abdullah Sheikh, Hesham S. Ghazzawy, Roshmon Thomas Mathew, Mohamed Ashour, Rania Ali El Hadi Mohamed, Ehab El-Haroun, Ali S. A. Saleem, Mohamed M. A. Abd-Elkarim

**Affiliations:** 1 Camel Research Center, King Faisal University, Al-Ahsa, Saudi Arabia; 2 Date Palm Research Center of Excellence, King Faisal University, Al-Ahsa, Saudi Arabia; 3 Fish Resources Research Center, King Faisal University, Al-Ahsa, Saudi Arabia; 4 Department of Biology, College of Science, Princess Nourah bint Abdulrahman University, Riyadh, Saudi Arabia; 5 Department of Integrative Agriculture, College of Agriculture and Veterinary Medicine, United Arab Emirates University, Abu Dhabi, United Arab Emirates; 6 Animal Production Department, Faculty of Agriculture, Sohag University, Sohag, Egypt; 7 Department of Animal Production, Faculty of Agriculture, Zagazig University, Zagazig, Egypt

**Keywords:** camel, methane emission, molecular docking, phytochemical extract, sustainable strategy

## Abstract

**Introduction:**

Sustainable management of bioactive-rich byproducts, including pomegranate peels, is crucial to support the rise of environmentally resilient intensive camel farming. The objective of this study was to investigate the impact of pomegranate peel extract (PPE) (0, 0.5, 1, and 2 g/kg) on methane production gas production, nutrient digestibility, and predictive values in camels through *in vitro* model and molecular docking simulations.

**Materials and methods:**

Rumen samples were fortified with PPE at levels of 0 (PPE0), 0.5 (PPE0.5), 1 (PPE1), and 2 (PPE2) g/kg diet to assess methane emissions, gas production, nutrient digestibility, and predictive values. Molecular docking was used to assess the inhibition of the methanogenic pathway enzymes formylmethanofuran dehydrogenase (Fmd), F420H2 oxidase, and shikimate dehydrogenase (SDH) by ellagic acid (EA) and punicalagin (PG).

**Results:**

The PPE1 and PPE0.5 groups showed significantly higher gas production across all incubation intervals (3, 6, 12, 24 and 48 h; *p* < 0.001). Supplementation at 0.5, 1, and 2 g/kg significantly (*p* < 0.05) lowered methane emissions (by 11.62, 13.17, and 19.39%) and total digestible dry matter (by 20.30, 22.19, 33.34%) relative to the control, respectively. PPE1 group significantly improved dry matter digestibility (*p* < 0.01, linear effect) and TVFA production (*p* < 0.05, quadratic effect) compared to the control. Rumen pH was significantly affected by treatment, with the lowest values observed in the PPE0.5 and PPE1 groups (*p* < 0.01, quadratic effect). All PPE groups had greater SCFA levels relative to the control group (*p* < 0.001, quadratic effect). The PPE1 and PPE0.5 treatments showed higher ME, NEL, and OMD, and lower partitioning factor (PF) compared to the other groups (*p* < 0.001, quadratic effect). The PPE1 group had the greatest MCP compared to other groups (*p* < 0.05). Docking analysis revealed that punicalagin (PG) exhibited superior binding affinities (−10.04 kcal/mol) against SDH compared to ellagic acid (EA), which reached a peak of −7.22 kcal/mol against F420H2 oxidase. PG also demonstrated better binding stability against F420H2 oxidase oxidase (−8.05 kcal/mol) compared to EA (−7.22 kcal/mol).

**Conclusion:**

These results suggest that dietary inclusion of 0.5 or 1 g/kg PPE significantly improves nutrient digestibility, productive performance, and rumen fermentation efficiency, while concurrently reducing methane emissions using an *in vitro* model in camels.

## 1 Introduction

Climate change and agricultural waste threaten livestock productivity by reducing feed and water availability, impairing animal health, and causing environmental pollution (1, 2). These challenges pose systemic threats to livestock stability, human health, and consumer demands (3, 4). Transforming agricultural byproducts into high-value commodities is essential for global environmental mitigation and the advancement of a circular bioeconomy (5). The dual challenge of ensuring global food security and mitigating the livestock sector’s 14% contribution to anthropogenic greenhouse gas emissions necessitates innovative management (5). Ruminants alone account for over 81% of enteric methane (CH_4_), a byproduct that not only traps heat more efficiently than CO_2_ but also wastes 2–12% of the animal’s energy intake (5, 6). Therefore, sustainable feeding strategies are needed to reduce emissions while maintaining animal productivity. In this context, agro-industrial byproducts have emerged as potent alternative feeds (7, 8), leveraging their unique bioactive compounds and nutritional density to reduce _CH4_ emissions while meeting the requirements of growing livestock populations.

Pomegranate (*Punica granatum* L.) is a high-value plant extensively cultivated in subtropical regions. Its peels, which make up 40–60% of the fruit’s weight, are a rich source of bioactive compounds such as catechins, phenolic acids, and tannins (9–12). Considering that peels account for 40–60% of the fruit’s total weight, this represents a substantial volume of raw material available for valorization. Pomegranate peels (PP) possess a complex chemical profile dominated by polyphenolic secondary metabolites, specifically hydrolysable tannins and punicic acid (13). Their bioactive content is diverse: they are abundant in various ellagitannins, such as ellagic acid (EA), hydrolysable tannins (HT), punicalagins, chebulagic acid, and mallotusinic acid, and further enriched by other health-promoting compounds (12, 14). This unique composition positions PP as an effective functional ingredient for animal nutrition, offering significant potential to optimize homeostatic stability, boost immune response, improve overall wellbeing and health (10, 15), and improve protein efficiency (16).

Gallic acid, a major component of HT, has been shown to reduce crude protein degradation *in vitro* by binding to feed proteins (17). Pomegranate tannins are known to be stable in the rumen (pH 5.0–7.0) and resistant to microbial degradation (18), while also exhibiting strong antioxidant activity (19). Daily supplementation of 100 or 200 g of dried PP per heifer may be beneficial for improving growth indices, immune function, and overall antioxidative status (20). Enteric methane (CH_4_) from ruminants is a major environmental concern due to its high global warming potential 28 times that of CO_2_, making the integration of phenol-rich feedstuffs a promising strategy for emission abatement (21–23). Research indicates that PPE reduces *in vivo* CH_4_ production in buffaloes while maintaining fermentation efficiency (24). These effects are primarily driven by active compounds, specifically tannins, which modulate rumen fermentation and inhibit methanogenic microorganisms (25). For instance, HT have been shown to suppress *in vitro* methanogen populations by approximately 12% (26), while the inclusion of pomegranate pomace at 15% of the diet reduced *in vitro* CH_4_ yield by 14% due to its richness in tannin compounds (27). Tannins act by directly targeting methanogens or reducing protozoal populations (28). Although PPE is known to improve fermentation, reduce CH_4_, and improve the health status of ruminants, research regarding its efficacy in camel nutrition, specifically through *in vitro* and *in silico* models, is currently lacking.

Camels are a cornerstone of pastoralist societies in arid and semi-arid ecosystems, with a global population of approximately 42 million (29). The genus *Camelus* comprises the one-humped dromedary (*Camelus dromedarius*) of the Middle East, Africa, and South Asia, and the two-humped Bactrian camel (*C. bactrianus*) of Central Asia (30). These species exhibit remarkable physiological resilience to climate change, maintaining productivity despite severe feed shortages, intense heat, and chronic water deficiency (29). In Egypt and other Arab regions, the camel represents a vital livestock resource, serving as a resilient provider of meat and milk in challenging environments. In these localities, camels support the livelihoods of numerous pastoralist communities facing increasing feed costs and environmental stressors. Recent nutritional interventions have shown promise in enhancing these traits; for instance, the inclusion of 2–4% date palm biochar optimized nutrient digestibility and fermentation while significantly reducing *in vitro* methane production (31). Similarly, phytogenic extract has been shown to improve hematological profiles and exert potent hepatoprotective, anti-inflammatory, and immunomodulatory effects in growing camels (32).

Network pharmacology integrates systems biology and multidirectional pharmacology to identify novel drug targets (33). This approach is highly effective for mitigating methane emissions by optimizing key enzymes in methanogenic pathways. In the livestock gut, *Methanobrevibacter* spp. are the primary methane-producing archaea, utilizing F420-dependent enzymes and formyl-methanofuran dehydrogenase (Fmd) to convert H_2_ and CO_2_ into methane (34–37). Recent metagenomic analyses of the camel rumen indicate that *Methanobrevibacter* species are the predominant methanogens (38). Furthermore, Shikimate 5-dehydrogenase (SDH) an NADP-dependent enzyme is essential for aromatic amino acid biosynthesis in bacteria (39). In silico molecular docking of gallic acid and punicalagin against these target enzymes facilitates the rapid screening of PPE-derived bioactives for their methane-mitigating potential.

Literature regarding the supplementation of PPE in camel diets is currently limited, with a lack of both *in vitro* and *in vivo* data. Given its richness in phytochemicals and biological activities of PPE, the study hypothesized that the dietary inclusion of PPE would enhance gas production, fermentation parameters, nutrient digestibility, and productive performance values while reducing methane emissions. Hence, this *in vitro* study, for the first time, aimed to explore the influence of PPE on gas production, methane emissions, nutrient degradability, fermentation parameters, and predicted camel performance using *in vitro* techniques and molecular analysis.

## 2 Materials and methods

All experimental work was conducted in the Laboratory of Animal Nutrition, situated within the Animal Production Department at the Faculty of Agriculture, Zagazig University, Zagazig, Egypt. The study protocols were approved by the ZU-IACUC committee (ZU-IACUC/2/F/25/2023). All animal care and experimental methods adhered to established ethical frameworks, including the U.K. Animals (Scientific Procedures) Act, 1986, EU Directive 2010/63/EU, the National Research Council’s Guide for the Care and Use of Laboratory Animals (NIH Publications No. 8023, revised 1978), and the ARRIVE guidelines.

### 2.1 Preparation of ethanolic pomegranate peel extract (PPE)

Pomegranate (*Punica granatum* L.) peels (PP) were obtained from a local market in Zagazig City, Sharkia Governorate, Egypt. The fruits are typically cultivated in the same region (El-Salhia, Sharkia), while the pomegranate processing facility is located in 10th of Ramadan City, Sharkia Governorate. Following collection, the PP were botanically authenticated by Professor El-Sayed El-Desoky, Department of Botany, Zagazig University, Faculty of Agriculture, to confirm the identity and taxonomic classification of the samples. The peel samples were rinsed with tap water followed by distilled water. After washing, the peels were manually removed, grounded into small pieces using a laboratory mill grinder (Retsch ZM 100, Retsch GmbH, Haan, Germany), and left to air-dry completely at room temperature. Ethanolic extracts were obtained by subjecting 100 g of pomegranate peel powder to Soxhlet extraction using 1 L of 80% ethanol as the solvent, following the method described in reference (10). The extracts were collected in amber bottles, filtered (45 μm), and evaporated under vacuum at 40 °C using a rotary evaporator (N-N series, EYELA) for 1–2 h. The 32% extraction yield corresponds to 32 g of extract per 100 g of pomegranate peel powder. The dried residues were stored at −18 °C until further use. The detailed phytochemical profile of the pomegranate peel extract (PPE) is summarized in Supplementary Table S1 (Supplementary Data), based on a comprehensive review of existing literature.

### 2.2 *In vitro* experiment design and chemical analysis

In this *in vitro* experiment, four experimental diets were formulated with varying levels of PPE inclusion for an *in vitro* study model. The first diet, designated as the PPE0 group, served as the basal control and was formulated without the addition of PPE, as detailed in Table 1. The remaining three experimental diets were based on the basal diet and fortified with PPE at inclusion levels of 0.5 (PPE0.5), 1.0 (PPE1), and 2.0 (PPE2) g/kg of diet. Pomegranate peel extract (PPE) was incorporated into the experimental diets during the mixing process to ensure a uniform mash formulation. The inclusion levels of PPE were selected based on those reported in previous studies (40, 41).

**Table 1:** The formulation and compound constituents of the basal diet used in this experiment.

Ingredients	Kg/100 kg
Wheat barn	12.5
Common salt	0.35
Yellow corn	44.8
Soybean meal	11.2
Limestone	0.80
Berseem hay	30.0
Mineral and vitamin mixture^a^	0.35
^a^Minerals and vitamins mixture contained: Copper 30,000 mg, iodine 800 mg, selenium 300 mg, iron 10,000 mg, MgO 80,000 mg, zinc 100,000 mg, cobalt 400 mg, Vit. A 10,000,000 IU, Vit. D_3_ 2,500,000 IU, Vit. E 35,000 IU, and CaCO_3_ to 3 Kg.	

Chemical composition (g/kg)	
Nutrient	Basal diet
Organic matter	940.6
Crude protein	140.4
Ether extract	30.5
Neutral detergent fiber	283.1
Acid detergent fiber	161.7
Ash	45.4
Crude fiber	123.6
Nitrogen-free extract	646.1

The basal experimental diet consisted of a mixture of 70% concentrate and 30% berseem hay (*Trifolium alexandrinum*). The diet, consisting of a 70:30 concentrate-to-berseem hay (*T. alexandrinum*) ratio, was milled using a laboratory mill grinder (Retsch ZM 100, Retsch GmbH, Haan, Germany). The ingredients were ground to pass through a 1-mm screen to ensure a uniform particle size suitable for *in vitro* fermentation. This substrate was subsequently analyzed for chemical composition and utilized in *in vitro* gas production assays. The ingredient formulation and chemical profile of the diet are presented in Table 1. The chemical composition of the experimental diets was determined using standard protocols (42): dry matter (DM; method 930.15), organic matter (OM; method 942.05), ether extract (EE; Soxhlet method 920.39), and crude protein (CP; Kjeldahl method 988.05). Ash content was determined by incinerating 2 g of the sample in a muffle furnace at 550 °C for 4 h. Fiber fractions, specifically neutral detergent fiber (NDF) and acid detergent fiber (ADF), were analyzed according to the procedures described by Van Soest et al. (43).

### 2.3 *In vitro* incubations

Ruminal fluid samples were collected from three slaughtered camels (mean body weight: 250 ± 8.66 kg) at a local slaughterhouse in Zagazig, Sharkia Governorate, Egypt, following the procedure described by Lutakome et al. (44). The camels originated from the Elshobaky Camel Farm (located near to Zagazig 5-6 km) and were fed the basal diet detailed in Table 1. Samples were collected in the early morning after the animals had been fasted for 12 h, with free access to water provided throughout the fasting period. The samples were transported to the Animal Nutrition Laboratory within 30–40 min of slaughter.

To maintain microbial viability, rumen fluid samples were (3 samples per animals, with totally nine ruminal samples) immediately transported to the laboratory in insulated containers maintained at 39 °C under strictly anaerobic conditions. The fluid was filtered through four layers of cheesecloth to remove large particulate matter and subsequently incubated in a 39 °C water bath. To ensure anaerobic conditions were preserved during preparation, the headspace of the fluid was purged with CO_2_ immediately prior to inoculation.

The buffered incubation medium (McDougall’s buffer, modified) consisted of NaCl (2.8 g/L), CaCl₂ (0.1 g/L), MgSO₄·7H₂O (0.1 g/L), Na₂HPO₄ (6 g/L), and KH₂PO₄·H₂O (2 g/L). The pH of the MB9 medium was adjusted to 6.8, and anaerobic conditions were maintained by flushing with CO₂ for 30 min (45).

The MB9 medium was mixed with the filtered rumen fluid at a 2:1 ratio (v/v). For incubation, glass tubes were used, each loaded with 200 mg of the experimental diet amended with pomegranate peel extract (PPE) at various concentrations. Each flask was inoculated with 30 mL of strained ruminal fluid (pre-warmed at 37 °C ± 2 °C), immediately sealed with a rubber stopper, and fitted with a three-way valve connected to a graduated plastic syringe for measuring gas production (31). Cumulative gas production was recorded at predetermined intervals (3, 6, 12, 24, 36, and 48 h) using calibrated glass syringes (46).

Each experimental run included four blank bottles (containing only the buffered rumen fluid, without substrate) to serve as negative controls, alongside six replicates for each dietary treatment. The final gas volume was recorded at the end of incubation. To estimate methane (CH_4_) emission, a 10 M NaOH solution was used to absorb CO_2_ from the gas, following the method of Fievez, Babayemi (47). Methane intensity was subsequently estimated and reported both as mL CH_4_ per truly degraded dry matter (TDDM) and as the percentage of CH_4_ within the total gas volume.

### 2.4 Assessment of ammonia-N, pH, and volatile fatty acids levels

Following each designated incubation interval, the tubes were opened, and the pH of the incubation medium was immediately measured using a calibrated digital pH meter (Model 6010 N; Jenco Instruments Inc., San Diego, CA, USA). To determine the TDDM, the procedure described by Blümmel et al. (48) was adopted. After 48 h of *in vitro* incubation, 30 mL of neutral detergent solution was added to three replicate tubes per treatment. The mixtures were then boiled, filtered through pre-weighed Gooch crucibles, and dried at 105 °C for 3 h to determine the weight of the truly undegraded residue. The remaining three replicate tubes per treatment were utilized for biochemical analysis. Total volatile fatty acid (TVFA) concentrations were determined via steam distillation Warner (49), while ruminal ammonia-nitrogen (NH_3_-N) concentrations were measured using the micro-diffusion technique Conway (50).

### 2.5 Calculations for partitioning factor, and nutrient degradation

The following parameters were determined for each treatment group: net energy of lactation (NEL, MJ/kg DM), metabolizable energy (ME, MJ/kg DM), *in vitro* organic matter digestibility (OMD, %), short-chain fatty acid (SCFA) concentrations, microbial crude protein (MCP) production, and the partitioning factor (PF). These variables were calculated based on the cumulative gas production and residual substrate data obtained from the three replicate tubes per treatment group.

- The net energy of lactation (NEL; MJ/kg DM) and metabolizable energy (ME; MJ/kg DM) were determined using the predictive equations established by Menke and Steingass (51), as follows:

$$\begin{array}{l} \;\mathrm{ME}\;(\frac{\mathrm{MJ}}{\mathrm{kg}\;\mathrm{DM}})=(0.157\times \mathrm{GP})+(0.0084\times \mathrm{CP})\\ +(0.022\times \mathrm{EE})-(0.0081\times \mathrm{CA})+1.06 \end{array}$$

$$\begin{array}{l} \mathrm{NEL}\;(\frac{\mathrm{MJ}}{\mathrm{kg}\;\mathrm{DM}})=(0.115\times \mathrm{GP})+(0.0054\times \mathrm{CP})\\ +(0.014\times \mathrm{EE})-(0.0054\times \mathrm{CA})-0.36 \end{array}$$

Where GP = net gas production (mL/0.2 g DM) at 24 h of incubation; EE, ether extract; CP, crude protein; CA, crude ash

- Short-chain fatty acid concentrations (SCFA) were calculated according to Getachew et al. (52) as:

$$\text{SCFA}\;(\frac{\text{mmol}}{200\mathrm{mg}\;\mathrm{DM}})=(0.0222\times \mathrm{GP})-0.00425$$

Where GP is the 24-h net gas production (mL/200 mg DM).

- Microbial crude protein production (MCP) was estimated according to Blümmel et al. (48) as follows:

$$\mathrm{MCP}(\frac{\mathrm{mg}}{g\;\mathrm{DM}})=\mathrm{mg}\;\mathrm{DMD}-(\;\mathrm{GP}\times 2.2)$$

Where GP is the 24-h net gas production (mL); 2.2 mg/mL is a stoichiometric factor that expresses mg of C, H, and O required to produce SCFA gas associated with the production of 1 mL of gas.

- The partitioning factor (PF, 24 h) was calculated according to the method of Blümmel et al. (48) as the ratio of truly degraded dry matter (TDDM, mg) to the volume of gas produced (GP, mL) at 24 h of incubation, expressed as follows:

$$\mathrm{PF}=\frac{\text{TDOM}}{\mathrm{GP}}$$

Where TDOM is total degradable organic matter (mg); GP is gas production volume (mL)

- The *in vitro* organic matter digestibility (OMD, %) was calculated according to the predictive equation developed by Menke et al. (53) as follows:

OMD(%)=14.88+(0.889×GP)+(0.45×CP)+(0.0651×CA)

Where GP = net gas production (mL/0.2 g DM) at 24 h of incubation; CP = crude protein; CA = Ash (%).

### 2.6 Molecular docking analysis

The potential affinities of Ellagic Acid and Punicalagin against three key Methanobrevibacter enzymes such as F420H(2) oxidase (UniProt ID: B1A7S3), Formylmethanofuran dehydrogenase (UniProt ID: A0A839NZW7), and Shikimate dehydrogenase (UniProt ID: A5UMF6) were determined using molecular docking approach.

Target protein structures were retrieved from the UniProt database, prepared by adding polar hydrogens, repairing atom valences, and subjected to energy minimization before being converted to PDBQT format. Ligand structures were constructed using ChemBioDraw Ultra 16.0, followed by the conversion of SDF files to PDBQT format, which included protonation and energy minimization (54). Molecular docking was performed using AutoDock Vina, and the binding energies (kcal/mol) for the most stable poses were recorded. The final docking conformations were analyzed and visualized using Discovery Studio 2024 to identify key intermolecular interactions.

### 2.7 Data analysis

The normality of data and homogeneity of variance were confirmed using the Shapiro–Wilk and Levene’s tests, respectively. Experimental data were analyzed using IBM^®^ SPSS^®^ software (version 21; Chicago, IL, USA). The statistical model used to evaluate the results was as follows:


$$Y_{\mathrm{ij}}=\mu +\alpha_{i}+e_{\mathrm{ij}}$$

Where *Y_ij_* is an observation, *μ* is the mean, *α*i is the treatment effect (pomegranate peel extract supplementation), and *e_ij_* is the standard error.

Significant differences among treatment means were evaluated using Duncan’s multiple range test. To assess the dose-dependent effects of PPE supplementation (0, 0.5, 1, and 2 g/kg of diet), orthogonal contrast statements (Linear and quadratic effects) were employed to determine the significance of linear and quadratic responses for all dependent variables. Differences among treatments were considered statistically significant at *p* < 0.05.

## 3 Results

### 3.1 Effect of PPE on gas production

Influence of graded levels of PPE on *in vitro* gas production parameters in camels are shown in Table 2. Adding 0.5 or 1 g of PPE to camel diets significantly improved gas production in a quadratic effect at 3, 6, 12, and 24 h of incubation (*p* < 0.001). There were no statistical differences between the high dose of PPE (2 g/kg diet) and the control diet at all time points of incubation (*p* > 0.05). A quadratic response was observed (*p* < 0.001), with the 1 g PPE inclusion reaching the highest gas production levels from 36 h through the end of the incubation period. At 36 h of incubation, no significant differences in gas production parameters were observed among the PPE0, PPE0.5, and PPE1 groups (*p* > 0.05). The PPE1 treatment resulted in the greatest gas production at 4 h, with PPE0.5 showing the next highest values (*p* < 0.01). Adding 0.5 g of PPE resulted in higher gas production compared to 2 g (PPE2 group) and the control group (PPE0.5) after 48 h of incubation (*p* < 0.001, quadratic effect).

**Table 2:** The effect of incorporating various dietary levels of pomegranate peel extract (PPE) on gas production parameters using the *in vitro* technique in camel diets.

Gas production (mL/g DM)	Treatment				*p*-value		
	PPE0	PPE0.5	PPE1	PPE2	ANOVA	Lin.	Quad.
3 h	19.58^b^ ± 1.76	37.92^a^ ± 2.77	36.25^a^ ± 2.72	26.25^b^ ± 2.21	<0.001	0.103	<0.001
6 h	45.42^b^ ± 1.64	56.67^a^ ± 5.43	63.33^a^ ± 2.79	42.50^b^ ± 2.81	0.001	0.894	<0.001
12 h	61.25^b^ ± 2.12	79.17^a^ ± 6.48	86.67^a^ ± 3.40	62.50^b^ ± 2.24	<0.001	0.533	<0.001
24 h	90.83^b^ ± 2.11	102.50^a^ ± 5.63	112.92^a^ ± 2.36	86.25^b^ ± 3.01	<0.001	0.836	<0.001
36 h	111.67^b^ ± 2.39	120.42^b^ ± 6.43	133.75^a^ ± 3.75	107.50^b^ ± 4.23	0.002	0.967	0.001
48 h	124.58^c^ ± 2.08	136.67^b^ ± 6.15	151.25^a^ ± 3.34	122.08^c^ ± 2.92	<0.001	0.691	<0.001

^a,b,c^Different letters indicate significant differences between groups (*p* < 0.05).

ANOVA, Lin., and Quad., orthogonal contrast statements were used to determine the significance of linear and quadratic responses for all dependent variables.

Results are expressed as mean ± standard error (SEM) (mean± SEM, *n* = 9).

The dietary treatments were: PPE0, PPE0.5, PPE1, and PPE2, corresponding to the basal diet (PPE0) or fortified with 0.5, 1, and 2 g of pomegranate peel extract/kg diet, respectively.

### 3.2 Effect of PPE on methane emission

The effects of various dietary levels of PPE on *in vitro* methane emission parameters in camel diets are presented in Table 3. Supplementing camel diets with PPE (0.5, 1, and 2 g) resulted in a significant reduction of methane emissions as mL/1 g DM with a linear effect (*p* < 0.001). Despite fortifying camel diets with PPE to reduce methane emissions expressed as mL/1 g TDDM, the lowest values of methane emissions were shown in the PPE2 group (linear effect, *p* < 0.001). Methane emissions, expressed as a percentage of total gas, were significantly reduced in a quadratic manner after the diet was fortified with 0.5, 1, and 2 g of PPE (*p* < 0.01).

**Table 3:** The effect of incorporating various dietary levels of pomegranate peel extract (PPE) on methane emission parameters using *the in vitro* technique in camel diets.

Item	Treatment				*p*-value		
	PPE0	PPE0.5	PPE1	PPE2	ANOVA	Lin.	Quad.
Methane emission							
mL/1 g DM	53.75^a^ ± 0.85	47.50^b^ ± 2.58	46.67^b^ ± 1.24	43.33^b^ ± 1.67	0.003	<0.001	0.404
mL/1 g TDDM	92.40^a^ ± 1.47	73.65^b^ ± 4.00	71.98^b^ ± 1.91	61.61^c^ ± 2.37	<0.001	<0.001	0.125
% of total gas	43.18^a^ ± 0.76	35.16^b^ ± 2.77	30.87^b^ ± 0.60	35.66^b^ ± 1.88	<0.001	0.003	0.001

^a,b,c^ Different letters indicate significant differences between groups (*p* < 0.05).

ANOVA, Lin., and Quad., orthogonal contrast statements were used to determine the significance of linear and quadratic responses for all dependent variables.

Results are expressed as mean ± standard error (SEM) (mean± SEM, *n* = 9).

The dietary treatments were: PPE0, PPE0.5, PPE1, and PPE2, corresponding to the basal diet (PPE0) or fortified with 0.5, 1, and 2 g of pomegranate peel extract/kg diet, respectively.

### 3.3 Effect of PPE on degradability and fermentation parameters

The effect of incorporating various dietary levels of PPE on degradability and fermentation parameters using the *in vitro* technique in camel diets is displayed in Table 4. The addition of PPE at a level of 1 g/kg in the camel diet increased dry matter digestibility (DMD) (*p* < 0.01, linear effect), while the levels of 0.5 and 2 g/kg diet had no significant effect on DMD compared to the control diet (*p* > 0.05). Adding PPE to the camel diet at 1 g exhibited the greatest level of DMD compared to the control diet, without significance among PPE-treated groups. However, there was no statistical difference among all supplemented groups (*p* > 0.01) and the control group for NH_3_-N (mg/100 mL). TVFA (mL/L) was the greatest in supplemented camel diets with 1 g of PPE compared to other groups (quadratic effect, *p* < 0.05). Rumen pH was significantly reduced in the PPE 0.5 group followed by the PPE 1 group with a quadratic effect (*p* < 0.01). All PPE groups were similar for DMD (*p* > 0.05). Groups PPE 2 and PPE 0.5 were similar for TVFA (mL/L) (p > 0.05). High levels of PPE did not produce a significant effect compared to the control diet (*p* > 0.05).

**Table 4:** The effect of incorporating various dietary levels of pomegranate peel extract (PPE) on degradability and fermentation parameters using the *in vitro* technique in camel diets.

Item	Treatment				*p*-value		
	Control	PPE0.5	PPE1	PPE2	ANOVA	Lin.	Quad.
Degradability							
DMD	58.17^b^ ± 1.17	64.83^ab^ ±2.05	70.33 ^a^ ± 2.18	64.50^ab^ ±2.46	0.019	0.004	0.842
Fermentation parameter							
NH_3_-N (mg/100 mL)	13.47 ± 1.07	15.87 ± 0.93	12.13 ± 2.47	12.13 ± 2.47	0.539	0.412	0.565
TVFA (mL/L)	94.67^b^ ± 8.01	103.00^b^ ± 6.11	128.33^a^ ± 3.18	105.33^b^ ± 3.18	0.013	0.794	0.022
pH	5.40^a^ ± 0.02	4.64^c^ ± 0.06	4.96^b^ ± 0.11	5.70^a^ ± 0.16	<0.001	0.015	<0.001

^a,b,c^Different letters indicate significant differences between groups (*p* < 0.05).

ANOVA, Lin., and Quad., orthogonal contrast statements were used to determine the significance of linear and quadratic responses for all dependent variables. TVFA, total volatile fatty acids, calculated as the sum of acetate, propionate, butyrate, isobutyrate, valerate, and isovalerate.

Results are expressed as mean ± standard error (SEM) (mean± SEM, *n* = 9).

The dietary treatments were: PPE0, PPE0.5, PPE1, and PPE2, corresponding to the basal diet (PPE0) or fortified with 0.5, 1, and 2 g of pomegranate peel extract/kg diet, respectively.

### 3.4 Effect of PPE on the predictive values

The production of short-chain fatty acids (SCFAs) significantly increased with the addition of PPE at levels of 0.5 and 1 g/kg diet, showing a quadratic effect (*p* < 0.001). The most notable enhancement was observed at a level of 1 g PPE/kg diet (Table 5). However, adding PPE at a high level (2 g/kg diet) did not show a substantial effect compared to the control diet. Camel diets supplemented with 1 or 0.5 g of PPE significantly improved the values of ME, NEL, and OMD compared to other groups in a quadratic effect (*p* < 0.001). The partitioning factor (PF, mg TDOM/mL gas) reached its lowest values in the PPE0.5 and PPE1 groups compared to the control and PPE2 treatments (*p* < 0.001, quadratic effect). Concurrently, microbial crude protein (MCP, mg/g DM) was significantly highest in the group supplemented with 1 g/kg of PPE, whereas supplementation at 0.5 g/kg and 2 g/kg did not result in significant differences compared to the control.

**Table 5:** The effect of incorporating various dietary levels of pomegranate peel extract (PPE) on predictive value parameters using the *in vitro* technique in camel.

Item	Treatment				*p*-value		
	Control	PPE0.5	PPE1	PPE2	ANOVA	Lin.	Quad.
SCFA (mmol)	0.40^c^ ± 0.01	0.45^b^ ± 0.02	0.50^a^ ± 0.01	0.38^c^ ± 0.01	<0.001	0.873	<0.001
ME (MJ/Kg DM)	4.06^b^ ± 0.07	4.43^a^ ± 0.18	4.76^a^ ± 0.07	3.92^b^ ± 0.09	<0.001	0.834	<0.001
NE_L_ (MJ/Kg DM)	1.82^b^ ± 0.05	2.09^a^ ± 0.13	2.33^a^ ± 0.5	1.72^b^ ± 0.07	<0.001	0.839	<0.001
OMD (%)	37.65^b^ ± 0.37	39.72^a^ ± 1.00	41.57^a^ ± 0.42	36.83^b^ ± 0.53	<0.001	0.837	<0.001
MCP (mg/g DM)	551.13^b^ ± 15.53	598.10^ab^ ±21.26	665.20^a^ ± 21.93	597.33^ab^ ±21.16	0.026	0.005	0.620
PF (mg TDOM/mL gas)	2.10^a^ ± 0.02	1.88^b^ ± 0.02	1.83 ^b^ ± 0.03	2.14^a^ ± 0.09	0.005	0.799	0.001

^a,b,c^Different letters indicate significant differences between groups (*p* < 0.05).

ANOVA, Lin., and Quad., orthogonal contrast statements were used to determine the significance of linear and quadratic responses for all dependent variables. SCFA, short-chain fatty acids, defined in this study as (the sum of acetate, propionate, and butyrate).

Results are expressed as mean ± standard error (SEM) (mean± SEM, *n* = 9). SCFA, short-chain fatty acids; ME, metabolizable energy; NEL, net energy lactation; MCP, microbial crude protein production; OMD, organic matter degradability; PF, partitioning factor.

The dietary treatments were: PPE0, PPE0.5, PPE1, and PPE2, corresponding to the basal diet (PPE0) or fortified with 0.5, 1, and 2 g of pomegranate peel extract/kg diet, respectively.

### 3.5 Molecular docking

Based on literature identifying ellagic acid and punicalagin as the major components of PPE, these molecules were utilized for molecular docking simulations. *Methanobrevibacter* spp. constitute a major portion of the methanogenic archaea population in camels (38). *Methanobrevibacter* spp. are the principal methane-producing archaea in the livestock gut, driving methanogenesis by converting H_2_ and CO_2_ via Fmd and F420-dependent enzymatic pathways.

**Figure 1:**
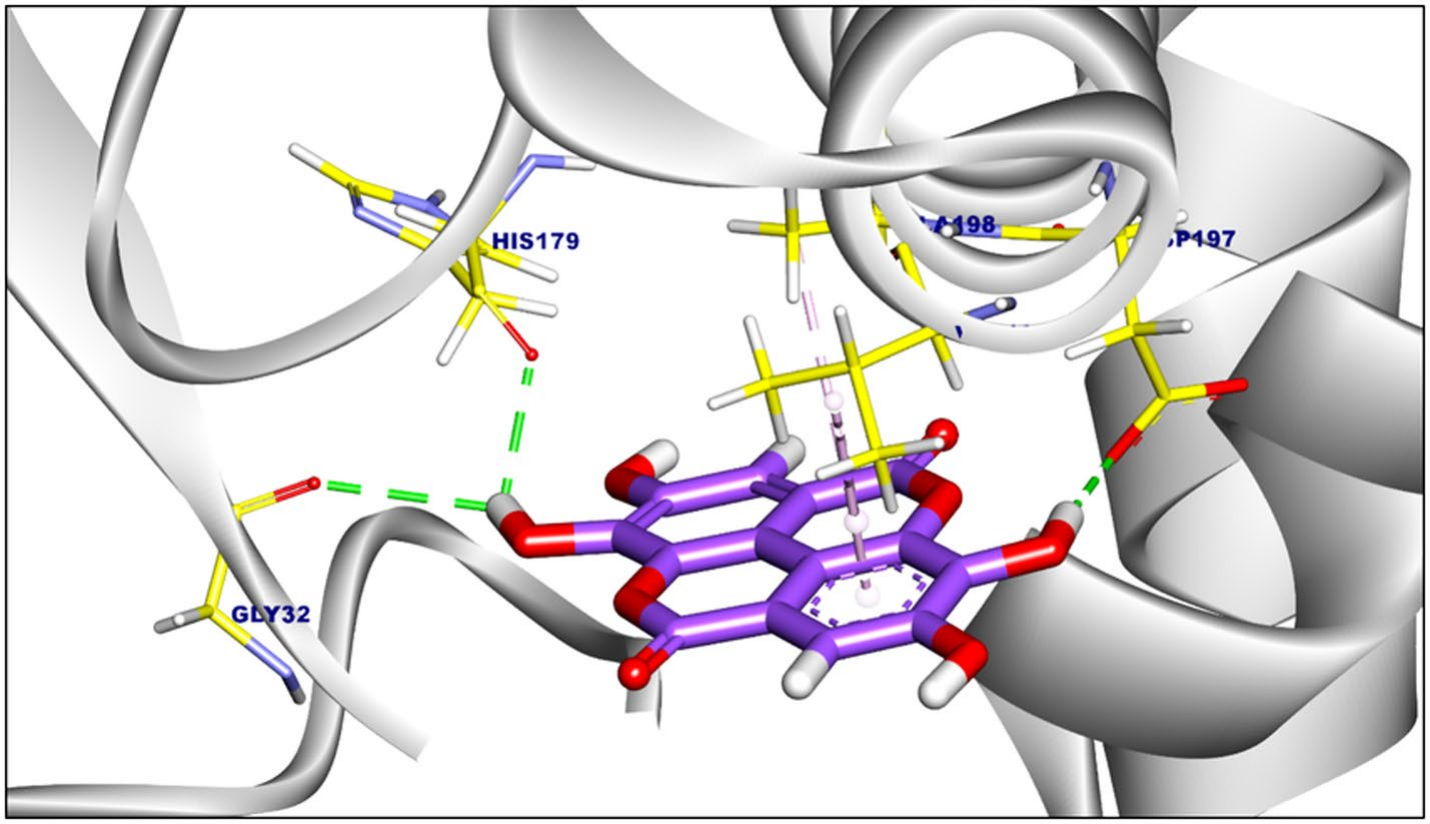
The three-dimensional representation illustrates the proposed binding of ellagic acid (violet) to F420H-oxidase, showing the interaction with critical yellow-colored amino acid residues in the binding pocket.

**Figure 2:**
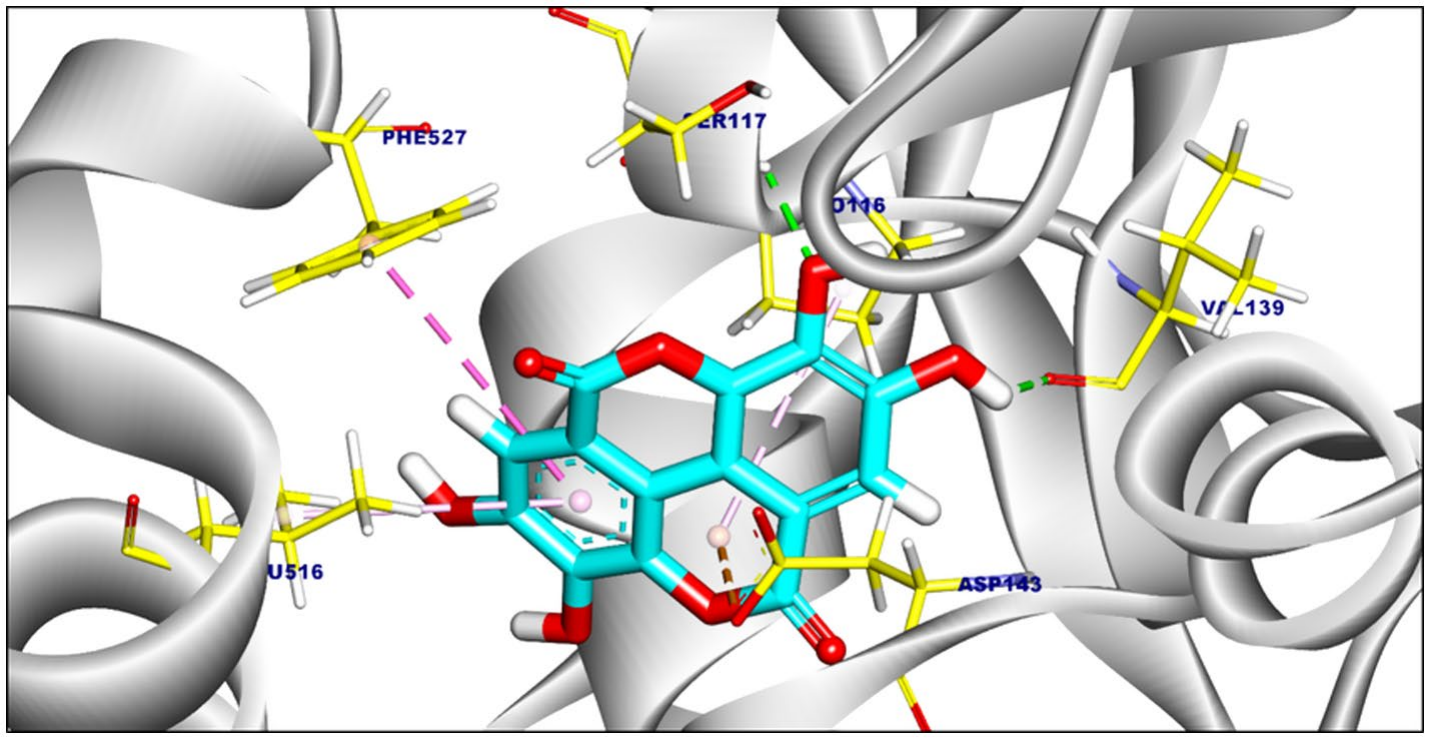
The 3D representation of the proposed binding mode of ellagic acid with formylmethanofuran dehydrogenase. Ellagic acid is shown in blue, and amino acid side chains are shown in yellow.

The binding affinities and interaction modes of ellagic acid and punicalagin against target enzymes were assessed through blind molecular docking (Table 6). Ellagic acid showed the highest affinity for F420H(2) oxidase (−7.22 kcal/mol; Figure 1) and formylmethanofuran dehydrogenase (−6.62 kcal/mol; Figure 2).

**Table 6:** Molecular docking analysis of ellagic acid and punicalagin against methanobrevibacter F420H2 oxidase, formylmethanofuran dehydrogenase, and shikimate dehydrogenase.

Tested targets	Compounds	RMSD value (Å)	Docking (affinity) score (kcal/mol)
F420H(2) oxidase	Ellagic acid	0.64	−7.22
	Punicalagin	1.61	−8.05
Formylmethanofuran dehydrogenase	Ellagic Acid	0.59	−6.62
Shikimate dehydrogenase	Punicalagin	1.83	−10.04

Three hydrophobic *π*-alkyl interactions were observed with Ala198 and Val194. Additionally, it formed three hydrogen bonds with His179 (2.32 Å), Gly32 (2.81 Å), and Tyr197 (2.59 Å) (Figure 1). Ellagic acid formed four hydrophobic interactions including *π*-alkyl, *π-π*, and *π*-anion interactions with Leu516, Asp143, Phe527, and Pro116 in Formylmethanofuran dehydrogenase. The interaction was supported by two hydrogen bonds with Ser117 (2.39 Å) and Val139 (1.98 Å) (Figure 2).

As the predominant and most bioactive constituent in PPE, punicalagin is a potent polyphenol recognized as the primary driver of pomegranate’s established biological activities (55). These include significant antioxidant, anti-inflammatory, antimicrobial, and antineoplastic properties.

**Figure 3:**
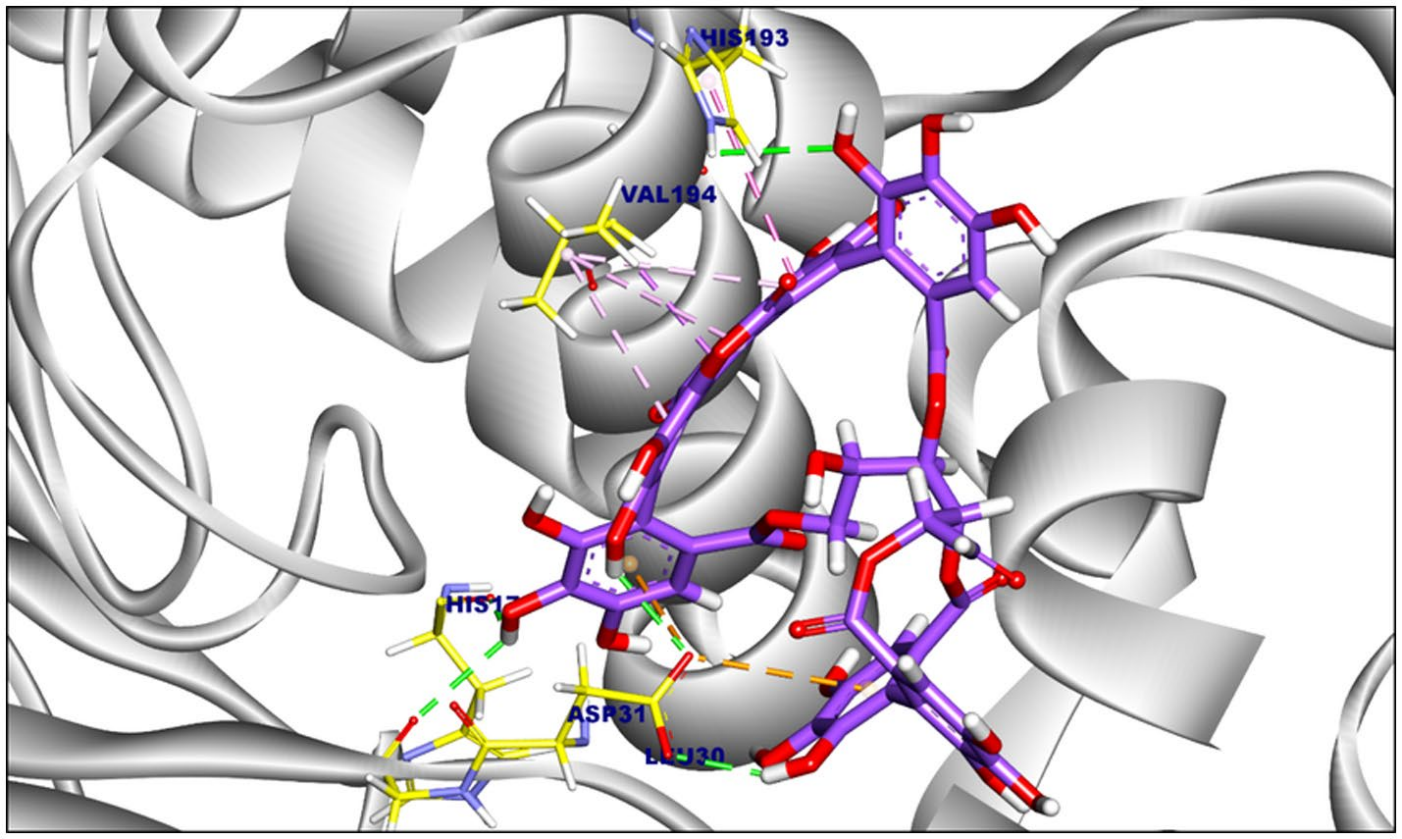
Proposed binding mode of punicalagin (PG) within the F420H2 oxidase active site. The highest-ranked docking pose of punicalagin is represented as violet sticks. Key amino acid residues within the catalytic pocket are illustrated in yellow, highlighting critical ligand-receptor interactions including hydrogen bonding and hydrophobic contacts.

**Figure 4:**
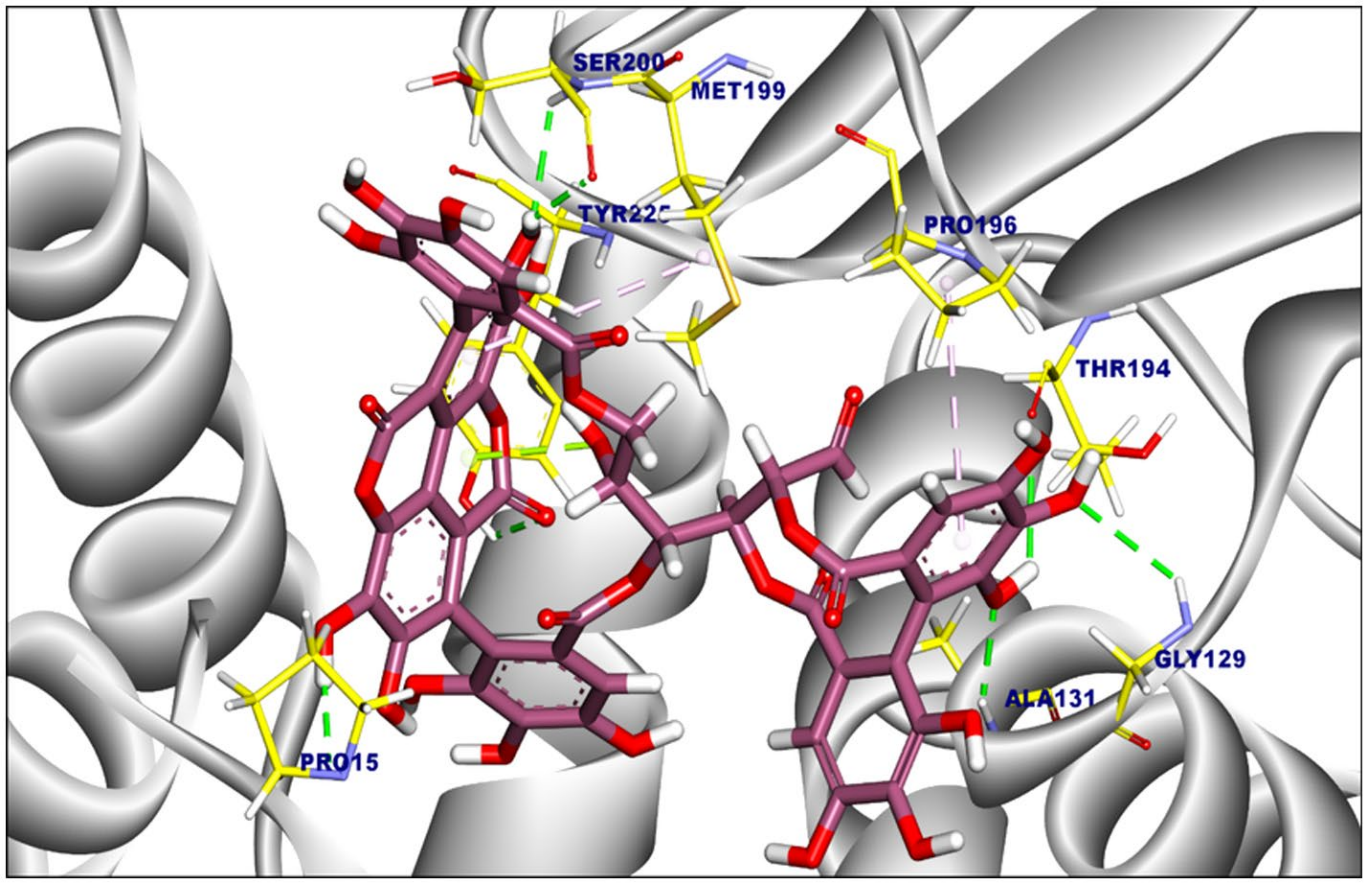
The proposed binding mode of punicalagin within the active site of shikimate dehydrogenase (SDH). Punicalagin is highlighted in blue, while the interacting amino acid side chains are depicted in yellow.

Punicalagin exhibited a binding affinity of −8.05 kcal/mol towards F420H2 oxidase oxidase, forming seven hydrophobic interactions (pi-alkyl, pipi, and pi-anion) with Asp31, Val194, and His193. Additionally, six hydrogen bonds were observed (Figure 3), with bond lengths ranging from 1.91 to 2.92 Å. Notably, punicalagin showed its highest affinity against shikimate dehydrogenase (−10.04 kcal/mol; Figure 4), established via seven hydrogen bonds with residues including Ser200, Tyr225, and Pro15.

## 4 Discussion

*In vitro* results indicated that dietary PPE inclusion significantly enhanced gas production (up to 48 h), nutrient degradability, and fermentation efficiency. Additionally, PPE improved predicted performance values (SCFA, NEL, OMD, and MCP synthesis) and consistently lowered methane emissions across all tested levels. Molecular docking demonstrated high binding affinities for key methanogenic enzymes, suggesting that the integration of PPE as a dietary byproduct is a promising strategy for enteric methane abatement. This approach provides a sustainable framework for the valorization of agro-industrial residues, simultaneously enhancing livestock productivity and mitigating environmental impacts.

Rumen gas production is influenced by a complex interplay between dietary composition, the resident microbial community, and the efficiency of fermentation-derived volatile fatty acid (VFA) utilization for microbial protein synthesis. The *in vitro* results of this experiment indicate that supplementing camel diets with 0.5 or 1 g of PPE significantly increased gas production via a quadratic effect at 3, 6, 12, 24 and 48 h of incubation compared to other groups. These observations align with existing literature regarding the impact of pomegranate peel constituents on improving gas kinetics. For example, Nezarati and Maheri-Sis (56) observed a significant, dose-dependent improvement in *in vitro* gas production when supplementing oilseed meals with PPE. Similarly, Kim et al. (57) reported an increase in total gas production by 5.2% in a diet supplemented with pomegranate extract compared to the control group. Additionally, Bayatkouhsar et al. (58) found that adding PPE led to higher total gas production compared to the basal diet. However, Abarghuei et al. (59) found that gas production at 24 h of incubation was not significantly affected by the type or level of PPE, possibly due to differences in dietary substrate composition, pomegranate extract method, and source of pomegranate. The increase in cumulative gas production observed with 0.5 or 1 g/kg PPE supplementation indicates significant beneficial effects on ruminal fermentation kinetics. These findings are primarily attributable to the high concentration of bioactive polyphenols, specifically ellagic acid and punicalagin, which likely modulate ruminal fermentation kinetics.

Plant secondary metabolites, especially phenolics, represent a viable strategy for enteric methane mitigation due to their antimicrobial properties. This study found that supplementation with PPE at 0.5, 1, and 2 grams resulted in a significant, dose-dependent linear reduction (*p* < 0.001) in methane emissions using various expressions. Relative to the control group, the 0.5, 1, and 2 grams of PPE reduced CH_4_ emissions by 11.62, 13.17, and 19.39% as emissions per gram of dry matter (mL/g DM), and 20.3, 22.19, and 33.34% as emissions per gram of total digestible dry matter (mL/g TDDM), respectively. The bioactive constituents of PPE enhance fermentation quality by favoring propionate production, which improves energy efficiency (9). Additionally, its antiprotozoal and antimicrobial effects (60), lower rumen ammonia levels and protozoal counts, thereby optimizing feed utilization and enhancing microbial protein synthesis (61). Methanogenesis involves the conversion of fermentation-derived H_2_ and CO_2_ into CH_4_ by archaea. Although necessary to prevent H_2_ accumulation and subsequent digestive inhibition (60), CH_4_ eructation represents a significant energy loss and environmental concern. Modulating this pathway is therefore vital for sustainable animal production. Interestingly, some rumen bacteria can utilize H_2_ to metabolize phenolic compounds like gallate and phloroglucinol, effectively diverting H_2_ away from methanogenesis and toward the synthesis of beneficial acetate (62). Experimental data support this mechanism: gallic acid, a phenolic monomer of hydrolysable tannins, has been shown to reduce archaeal abundance and H_2_ accumulation while simultaneously stimulating the production of acetate and butyrate (63). A study by Bayatkouhsar et al. (58) demonstrated that extracts from various sources, including *Punica granatum*, significantly reduced *in vitro* CH_4_ emissions. These results align with findings by Stewart et al. (64) regarding the efficacy of tannin-containing forages in lowering CH_4_ output in beef cattle. Collectively, these studies suggest that the observed reduction in CH_4_ is likely mediated by the selective inhibitory effects of tannins on key methanogenic microbial populations (65).

Tannins specifically suppress ruminal protozoal populations, which Morgavi et al. (66) identified as a key factor in enteric CH_4_ production. Furthermore, the inhibition of certain fibrolytic bacteria, the primary producers of the H_2_ utilized by methanogens, offers an additional pathway for CH_4_ suppression. While these modulatory effects can enhance metabolic energy output, the antimicrobial nature of tannins means their impact on total gas volume remains variable, often resulting in reduced production at higher inclusion levels. Incorporating phyto-molecules, such as tannins and saponins, represents a multifaceted strategy for CH_4_ mitigation. These compounds function through several mechanisms: the direct inhibition of methanogens and protozoa, or the modification of fermentation pathways to favor propionate formation, which acts as a hydrogen-consuming sink (67). Additionally, specific phytomolecules serve as non-methanogenic electron acceptors, effectively diverting available H_2_ away from methanogenesis (68).

Enhancing nutrient degradability and rumen fermentation parameters through bioactive compounds can increase microbial protein synthesis and energy availability, thereby improving the overall productivity and physiological status of ruminants. The *in vitro* results demonstrated that supplementation with 1 g/kg PPE significantly improved dry matter digestibility (DMD) and total volatile fatty acid (TVFA) concentrations, without adversely affecting ruminal ammonia-nitrogen (NH_3_-N) levels (*p* > 0.05). The impact of PPE on rumen digestibility shows considerable variation across the literature. For instance, Jami et al. (69), and Abarghuei et al. (70) demonstrated that PPE inclusion (1–4% of the diet) improved the digestibility of DM, CP, and neutral detergent fiber (NDFom) in dairy cows. In contrast, Oliveira et al. (71) reported that feeding dried pomegranate extract decreased whole-tract fat and CP digestibility in calves, likely due to the higher tannin content. Furthermore, other studies have observed no significant impact on apparent DM and OM digestibility when using high inclusion levels (20–30% of DM) (72). Overall, these inconsistencies are likely attributable to differences in the concentration and chemical structure of the tannins, as well as the variability in the basal diet composition (25). As ruminal VFAs serve as the primary energy source for ruminants, dietary strategies that optimize VFA production are critical for enhancing animal health and overall productivity. Evidence from Abarghuei et al. (70) indicated that supplementing dairy cows with PPE reduced ruminal protozoal populations and NH_3_N concentrations while simultaneously enhancing microbial protein synthesis. In a related *in vitro* study, Abarghuei et al. (59) demonstrated that PPE effectively modulates ruminal fermentation kinetics in sheep, specifically shifting the volatile fatty acid profile by increasing propionate concentration while concurrently reducing acetate, NH_3_N generation, and protozoal density. These metabolic shifts underscore the potential of PPE as a tool to enhance energy availability in the rumen. Several studies have reported that the inclusion of plant extracts rich in saponins and tannins influences ruminal pH. The results showed that supplementation with 0.5 or 1 g/kg of PPE significantly reduced ruminal pH values compared to the PPE at 2 g/kg (PPE2) and control groups (*p* < 0.05). These results are corroborated by previous studies that reported similar fermentation patterns, as observed in our current experiment. For instance, Min et al. (73) observed that supplementation with *Lotus corniculatus* (containing 3.2% condensed tannins) reduced ruminal pH in sheep. Similarly, Bayatkouhsar et al. (58) found that both aqueous and alcoholic *P. granatum* peel extracts reduced *in vitro* rumen pH. Conversely, other studies reported no significant impact of *P. granatum* extract on the rumen pH of sheep (72) or dairy cows (70). One primary mechanism for pH reduction is a shift in the microbial community, specifically the inhibition of cellulolytic bacteria (58, 74). Additionally, the net effect on pH is dependent on the temporal relationship between feed consumption and the rate of VFA production (75). Variations in tannin concentration, extract type, and basal diet composition further contribute to the inconsistent results observed across different experimental models.

The Partitioning Factor (PF) is a crucial metric for evaluating nutrient partitioning during rumen fermentation. It is defined as the ratio of truly degraded organic matter (TDOM, mg) to the total volume of gas produced (mL). The PF reflects the efficiency with which the substrate is converted into SCFAs, microbial biomass, and fermentation gases. A higher PF indicates enhanced microbial protein synthesis (MCP) and a more efficient conversion of nitrogen from degraded organic matter into microbial biomass (76). The *in vitro* experiment showed that adding PPE (0.5 or 1 g) at different levels significantly enhanced SCFA, ME, NEL, MCP, and OMD.

Research regarding the influence of herbal additives on the PF has yielded inconsistent results. For instance, certain studies report that saponin-containing additives reduce the PF by approximately 5.8% relative to control diets. Conversely, Benaouda et al. (77) observed a significant increase in the PF during the incubation of tannin-rich forages, whereas Castro-Montoya et al. (78) found that the PF remained unaffected by the supplementation of either tannins or saponins. Additionally, Bayatkouhsar et al. (58) demonstrated that aqueous and methanolic extracts of *Camellia sinensis*, *P. granatum*, and *Quercus persica* decreased the PF only at high concentrations (100 and 200 mg/mL), suggesting that the impact of tannins on nutrient partitioning is highly dependent on both the type and concentration of the extract (79).

Tannins and other plant-derived bioactive compounds exert a complex, dose-dependent influence on microbial crude protein production (MCP), a critical driver of ruminant productivity. Research indicates that the impact of tannins on MCP efficiency is highly sensitive to dosage: low inclusion levels (e.g., 50 mg/mL) have been shown to enhance MCP efficiency, whereas higher concentrations (e.g., 200 mg/mL) can suppress it (58). Both condensed and hydrolyzable tannins regulate MCP by modulating nitrogen dynamics in the rumen, notably by protecting dietary protein from extensive ruminal degradation. However, this protection must be balanced against the potential reduction in overall nutrient availability. Tannins such as found in PPE effectively redirect nutrient utilization, shifting the partitioning of substrate toward microbial biomass at the potential expense of SCFA production (77, 80). Consequently, identifying the optimal inclusion level is essential to maximizing nitrogen utilization efficiency without compromising total energy yield. Low concentrations of tannins may optimize ruminal fermentation by slowing the rate of dietary protein degradation, thereby enhancing the synchronization of nitrogen and energy release (18). This temporal alignment of substrate availability is critical for maximizing the efficiency of MPS. Furthermore, the selective inhibition of ruminal protozoa and bacteriophages by tannins contributes positively to the net yield of MCP (78). It could be suggested that low to moderate levels, tannins act as effective modulators of rumen fermentation, improving the overall efficiency of microbial protein synthesis by protecting protein and synchronizing nutrient availability.

Short-chain fatty acids (SCFAs) represent the primary energy source for ruminants, generated via microbial fermentation within the rumen. Our study found that PEE at 1 g/kg significantly increased SCFA levels compared to all other groups. Additionally, the 0.5 g/kg dosage resulted in higher SCFA levels than the 2 g/kg and control groups. Similar to our result, a study by Bayatkouhsar et al. (58) observed increased SCFA levels following PPE supplementation compared to control diets. Conversely, Abarghuei et al. (70) reported no significant changes in total SCFA concentrations under similar conditions. This variability extends to other tannin-rich sources; while purified tannins from *Quercus incana* and *Dichrostachys cinerea* have been shown to reduce SCFA production (80), or supplementation of soybean meal with tannins in Holstein bulls yielded no significant effects (81). Such inconsistencies may be attributed to the specific phenolic profile, tannin concentrations, and other bioactive compounds present in the PPE, alongside variations in the basal diet composition used across studies.

Our study provides mechanistic evidence of their inhibitory effects on key enzymes in Methanobrevibacter spp. *In silico* molecular docking demonstrated high binding affinities between PPE bioactives and key methanogenic enzymes, suggesting that the integration of this agro-industrial byproduct into ruminant diets is a potent strategy for enteric methane abatement. EA mitigates CH_4_ production by suppressing methanogens, specifically Methanobrevibacter sp. Abm4. This decline reduces competitive pressure for H_2_, increasing its availability for more energy-efficient pathways. By diminishing the methane hydrogen sink, EA facilitates a metabolic shift toward hydrogen-consuming bacteria involved in propionate synthesis. Consequently, this redirection of metabolic precursors optimizes fermentation end-product utilization and enhances ruminal energy capture (82).

This observation is consistent with broader research indicating that tannins, the primary agents responsible for inhibiting ruminal methanogenesis, suppress methanogenic growth. Although both hydrolysable and condensed tannins are known to modulate methanogen activity, lower dosages are particularly effective at inhibiting CH_4_ production through synergistic antimethanogenic and antiprotozoal activities (82). This suppression can result in a direct decrease in methanogenic archaea populations or a simultaneous reduction in both archaea and their protozoal hosts (25). Targeting methane emission pathways through enzymatic inhibition represents an innovative approach to reducing enteric gas emissions (83, 84), providing a scalable strategy to mitigate the livestock sector’s contribution to global warming. By disrupting the specific enzymes essential for the methanogenesis pathway, these phytochemicals offer a precise intervention to shift ruminal fermentation towards more sustainable outcomes.

Several limitations of the present study should be acknowledged. First, the *in vitro* nature of the fermentation assays may not fully replicate the complex physiological conditions found *in vivo*. Additionally, the relatively small sample size may limit the broader generalizability of these findings. Furthermore, the absence of quantitative phytochemical profiling via HPLC-DAD or LC–MS/MS precludes a direct correlation between specific PPE bioactives and the observed microbial shifts. Future research incorporating high-resolution metabolomic profiling is essential to comprehensively map these biochemical interactions.

## 5 Conclusion

The *in vitro* assessment revealed that the inclusion of pomegranate peel extract (PPE) at 0.5 or 1 g/kg diet significantly optimized fermentation kinetics, specifically enhancing cumulative gas production (up to 48 h), nutrient degradability, and overall fermentation efficiency. PPE (0.5 or 1 g/kg) supplementation also improved key predictive performance indicators, including short-chain fatty acid (SCFA) profiles, net energy for lactation (NEL), organic matter digestibility (OMD), and microbial crude protein (MCP) synthesis. Concurrently, methane emissions were consistently suppressed across all inclusion levels. Complementary molecular docking analysis demonstrated high binding affinities between PPE bioactives and essential methanogenic enzymes, reinforcing the potential of this agro-industrial byproduct as a dual-purpose strategy for enteric methane abatement and enhanced livestock productivity. Future research incorporating high-throughput approaches, such as nutrigenomics and proteomics, will be essential to fully elucidate the underlying molecular mechanisms and metabolic interactions.

## Data Availability

The original contributions presented in the study are included in the article/Supplementary material, further inquiries can be directed to the corresponding authors.

## Glossary

PP: pomegranate peels
PPE: pomegranate peel extract
PG: punicalagin
Fmd: formylmethanofuran dehydrogenase
SDH: shikimate dehydrogenase
EA: ellagic acid
HT: hydrolysable tannins
CP: crude protein
CH_4_: methane
GHG: greenhouse gases
DM: dry matter
OM: organic matter
EE: ether extract
NDF: neutral detergent fiber
ADF: acid detergent fiber
TDDM: truly degraded dry matter
TVFA: total volatile fatty acid
NEL: net energy of lactation
ME: metabolizable energy
OMD: *in vitro* organic matter digestibility
SCFA: short-chain fatty acid
MCP: microbial crude protein
PF: partitioning factor
MPS: Microbial protein synthesis
