# Supplementary material for: Dose-dependent effects of pomegranate peel extract on modulating ruminal fermentation, methane emission, nutrient digestibility and productive values in camels: an in vitro and in silico integrations
Source: Front Vet Sci. 2026 May 4;13:1769637. doi: 10.3389/fvets.2026.1769637 (PMC13180871; doi:10.3389/fvets.2026.1769637)
Supplement: Supplementary file 1 [file Table_1.DOCX]

**Table S1.** The major compounds identified in PEP, based on existing literature, are summarized below

| **Compounds** | **Concentration** | **Chemical Formula** | **Reference** |
| --- | --- | --- | --- |
| **Gallic Acid** | 32.2 mg/g | C_7_H_6_O_5_ | Kumar et al., 2022 |
|  | 123.79mg/100g |  | Elfalleh et al., 2011 |
| **Ellagic Acid** | 13.6 mg/g | C_14_H_6_O_8_ | Kumar et al., 2022 |
|  | 35.89 mg/100g |  | Elfalleh et al., 2011 |
| **Caffeic acid** | 20.56 mg/100 g |  | Elfalleh et al., 2011 |
| **Punicalagin** | 15.2 mg/g | C_48_H_28_O_30_ | Kumar et al., 2022 |
|  | 16.67–245.47 mg/g DM |  | Aqil et al., 2012 |
| **Quercetin** | 2.5 mg/g | C_15_H_10_O_7_ | Kumar et al., 2022 |
| **Tannins** | 193 - 420 mg/g |  | Tang et al., 2010 |
| **Flavonoids** | 84, - 134 mg/g DM |  | Tang et al., 2010 |

**References**

Aqil F, Vadhanam MV, Gupta RC. Enhanced activity of punicalagin delivered via polymeric implants against benzo[a]pyrene-induced DNA adducts. Mutat Res. (2012) 743:59–66. 10.1016/j.mrgentox.2011.12.022

Azmat, F., Safdar, M., Ahmad, H., Khan, M. R. J., Abid, J., Naseer, M. S., ... & Suleria, H. A. R. (2024). Phytochemical profile, nutritional composition of pomegranate peel and peel extract as a potential source of nutraceutical: A comprehensive review. *Food Science & Nutrition*, *12*(2), 661-674.‏

Kumar N, Pratibha, Neeraj, Sami R, Khojah E, Aljahani AH, Al-Mushhin AAM. Effects of drying methods and solvent extraction on quantification of major bioactive compounds in pomegranate peel waste using HPLC. Sci Rep. 2022 May 14;12(1):8000. doi: 10.1038/s41598-022-11881-7. PMID: 35568719; PMCID: PMC9107484.

Elfalleh W, Tlili N, Nasri N, Yahia Y, Hannachi H, Chaira N, et al. Antioxidant capacities of phenolic compounds and tocopherols from Tunisian pomegranate (Punica granatum) fruits. *J Food Sci.* (2011) 76:C707–13. 10.1111/j.1750-3841.2011.02179.x

Tang L, Liu L, Sun L, Qin Y, Li J, Li X. Extraction and composition analysis of polyphenols in pomegranate skin. *J Food Res Dev.* (2010) 05:121–6.
